# Characterization and Identification of Subpopulations of Mononuclear Preosteoclasts Induced by TNF*-*α in Combination with TGF-β in Rats

**DOI:** 10.1371/journal.pone.0047930

**Published:** 2012-10-24

**Authors:** Rei Matsubara, Toshio Kukita, Yuka Ichigi, Ippei Takigawa, Peng-Fei Qu, Noboru Funakubo, Hiroshi Miyamoto, Kazuaki Nonaka, Akiko Kukita

**Affiliations:** 1 Department of Microbiology, Faculty of Medicine, Saga University, Nabeshima, Saga, Japan; 2 Pediatric Dentistry, Faculty of Dental Science, Kyushu University, Maidashi, Fukuoka, Japan; 3 Molecular Cell Biology and Oral Anatomy, Kyushu University, Maidashi, Fukuoka, Japan; 4 Oral and Maxillofacial Oncology, Kyushu University, Maidashi, Fukuoka, Japan; University of Medicine and Dentistry of New Jersey, United States of America

## Abstract

Osteoclasts are unique multinucleated cells formed by fusion of preosteoclasts derived from cells of the monocyte/macrophage lineage, which are induced by RANKL. However, characteristics and subpopulations of osteoclast precursor cells are poorly understood. We show here that a combination of TNF*-*α, TGF-β, and M-CSF efficiently generates mononuclear preosteoclasts but not multinucleated osteoclasts (MNCs) in rat bone marrow cultures depleted of stromal cells. Using a rat osteoclast-specific mAb, Kat1, we found that TNF*-*α and TGF-β specifically increased Kat1^+^c-fms^+^ and Kat1^+^c-fms^−^ cells but not Kat1^−^c-fms^+^ cells. Kat1^−^c-fms^+^ cells appeared in early stages of culture, but Kat1^+^c-fms^+^ and Kat1^+^c-fms^−^ cells increased later. Preosteoclasts induced by TNF*-*α, TGF-β, and M-CSF rapidly differentiated into osteoclasts in the presence of RANKL and hydroxyurea, an inhibitor of DNA synthesis, suggesting that preosteoclasts are terminally differentiated cells. We further analyzed the expression levels of genes encoding surface proteins in bone marrow macrophages (BMM), preosteoclasts, and MNCs. Preosteoclasts expressed *itgam* (CD11b) and chemokine receptors CCR1 and CCR2; however, in preosteoclasts the expression of chemokine receptors CCR1 and CCR2 was not up-regulated compared to their expression in BMM. However, addition of RANKL to preosteoclasts markedly increased the expression of CCR1. In contrast, expression of macrophage antigen *emr-1* (F4/80) and chemokine receptor CCR5 was down-regulated in preosteoclasts. The combination of TNF-α, TGF-β, and M-CSF induced Kat1^+^CD11b^+^ cells, but these cells were also induced by TNF-α alone. In addition, MIP-1α and MCP-1, which are ligands for CCR1 and CCR2, were chemotactic for preosteoclasts, and promoted multinucleation of preosteoclasts. Finally, we found that Kat1^+^c-fms^+^ cells were present in bone tissues of rats with adjuvant arthritis. These data demonstrate that TNF*-*α in combination with TGF-β efficiently generates preosteoclasts *in vitro*. We delineated characteristics that are useful for identifying and isolating rat preosteoclasts, and found that CCR1 expression was regulated in the fusion step in osteoclastogenesis.

## Introduction

Osteoclasts are bone-resorbing multinucleated cells that originate from multipotent hematopoietic stem cells in bone marrow [1], [2]. Early osteoclast precursor cells originate from cells of the monocyte/macrophage lineage. Osteoclast precursor cells differentiate into mononuclear preosteoclasts. Differentiated preosteoclasts fuse to form multinucleated osteoclasts. Osteoclasts are then activated to become mature osteoclasts having bone resorption activity. Receptor activator of NF-κB ligand (RANKL) and macrophage-colony-stimulating factor (M-CSF) are essential factors driving differentiation into mature osteoclasts from osteoclast precursors [3], [4]. These two factors bind to their respective receptors, RANK and c-fms, on the osteoclast precursor cell surface, and activate downstream signals such as Fos and nuclear factor of activated T cells c1 (NFATc1) [5]. The addition of RANKL with M-CSF to *in vitro* cultures of bone marrow cells or bone marrow macrophages (BMM) sequentially induces their differentiation into preosteoclasts and then to osteoclasts. However, because the culture contains osteoclasts, preosteoclasts, and their precursor cells, it is difficult to analyze characteristics of osteoclast precursor cells, *per se.*

Fusion of preosteoclasts during osteoclastogenesis is a critical process for bone resorption. It has been shown that several membrane proteins including CD9, cadherin, a member of the ADAM (disintegrin and metalloenzyme) family, and DC-STAMP (dendritic cell-specific transmembrane protein) are involved in multinucleation in osteoclastogenesis [6], [7], [8], [9]. Gene targeting of DC-STAMP in mice results in markedly increased bone mass because of impaired osteoclast fusion. On the other hand, recent findings indicate that CC chemokine receptors CCR1 and CCR2 are involved in fusion during osteoclastogenesis. Osteoclastognesis from CCR1 knockout mice was impaired due to a defect in multinucleation [10], and a CCR1 inhibitor MLN3897 inhibited the fusion of human osteoclast precursor cells [11]. We and others previously reported that CCR1 and CCR2 ligands such as macrophage inflammatory protein-1α (MIP-1α) and monocyte chemoattractant protein-1 (MCP-1) stimulated RANKL-induced differentiation and fusion during osteoclastogenesis [12], [13]. Parathyroid hormone induced expression of MCP-1 in osteoblasts, which promoted fusion of osteoclasts [14]. However, the mechanism of osteoclast fusion remains unclear. It is also not known how the expression of these genes is regulated during differentiation of precursor cells into multinucleated cells.

Cell surface proteins are useful for identifying preosteoclasts. It has been shown that the phenotype of early-stage mouse osteoclast precursor cells is c-fms^+^/RANK^−^ [15]. M-CSF stimulated these precursor cells to differentiate into c-fms^+^/RANK^+^ late osteoclast precursor cells, which can then differentiate into osteoclasts in the presence of M-CSF and RANKL. However, BMM are also c-fms^+^/RANK^+^, and detailed characteristics of c-fms^+^/RANK^+^ osteoclast precursor cells are not well known. We have established mAb Kat1, which recognizes a surface antigen expressed on osteoclasts in rat bone marrow culture systems and *in vivo* [16], [17]. We have reported that Kat1 antigen is specifically expressed by cells of the osteoclast lineage, and formation of Kat1^+^ cells was consistent with osteoclast differentiation [18], [19], [20], [21], [22]. In addition, expression of Kat1 antigen closely correlated with that of the calcitonin receptor in preosteoclasts and osteoclasts. Kat1 antibody reacted not only to osteoclasts formed *in vitro* but also osteoclasts *in situ* [17], [23].

Previous studies have shown that TNF*-*α induced osteoclast formation from RANKL-primed or TGF-β-primed mouse BMM in a RANKL-RANK independent manner *in vitro* [24], [25]. We have previously developed a rat culture system in which preosteoclasts are formed from bone marrow cells deprived of stromal cells [20], [26]. In the culture system, TNF*-*α enhanced the formation of preosteoclasts in the presence of osteoblast conditioned medium, but TNF*-*α alone did not induce the formation of preosteoclasts [19]. In the current study, we show that TNF*-*α in combination with TGF-β in the presence of low concentrations of M-CSF efficiently generates preosteoclasts that express osteoclast-specific genes and Kat1 antigen *in vitro*. In addition, we found that preosteoclasts induced by TNF*-*α and TGF-β represented a late stage of differentiation, and we found that expression of CCR1 was not induced in preosteoclasts, but was induced during multinucleation.

## Materials and Methods

### Animals and Chemicals

FCS was purchased from BioWhittaker (Walkersville, MD). Recombinant human sRANKL, M-CSF, and OPG were from Pepro-Tech (London, United Kingdom). Recombinant rat TNF*-*α and human TGF-β were purchased from Boehringer Mannheim (Mannheim, Germany) and R&D Systems (Minneapolis, MN), respectively. Rabbit anti-fms/CSF-1 receptor antibody was obtained from Upstate (Billerica, MA). Unlabeled anti-rat CD11b/c and Alexa Flour 488 mouse anti-rat CD11b/c (OX-42) were obtained from Biolegend (San Diego, CA). Mab Kat1 was prepared in our laboratory as described previously [17]. Biotin labeling kit was obtained from GE Healthcare. Goat anti-rabbit Alexa Fluor 488 and anti-mouse Alexa Fluor 568 antibodies were obtained from Molecular Probes. FITC-conjugated Streptavidin was obtained from eBioscience (San Diego, CA). MIP-1α, and MCP-1 were obtained from Invitrogen (CA, Carlsbad). Male Sprague Dawley (SD) rats (aged 7–10 weeks) were purchased from Kyudo Co. (Saga, Japan).

### Ethics Statement

All experiments were reviewed and approved by the Laboratory Animal Care and Use Committee of Saga University, permit number (21-006-0), and Kyushu University, permit number (A23-083-0).

### Formation of BMM, Preosteoclasts, and Osteoclasts (MNCs) in Rat Bone Marrow Cultures

Cultures of rat bone marrow cells were carried out as described by Kukita *et al.* [26]. Briefly, stromal cells were depleted by passage through a Sephadex G-10 column, and the nonadherent bone marrow cells (NABMCs) were recovered. To form preosteoclasts, NABMCs were cultured in the presence of various concentrations of TNF*-*α, TGF-β, and M-CSF for 2 to 3 days. In some experiments, combinations of RANKL, MIP-1α, and MCP-1 were added after 2 days of culture, and cells were cultured for an additional 2 to 3 days to induce MNCs. To form BMM, NABMCs were cultured in the presence of M-CSF (3 ng/ml) for 3 days. At the end of the culture, the cells were fixed and stained with a commercial kit for the osteoclast marker tartrate-resistant acid phosphatase (TRAP) (Sigma). TRAP^+^ cells with 3 or more nuclei were counted as MNCs. For immunostaining for Kat1 mAb, bone marrow cells were cultured in the presence of 10^−8^ M 1,25(OH)_2_D_3_ to form osteoclasts as described [27].

### RT-PCR and Real-time RT-PCR

Total RNA was extracted using Isogen (Nippon Gene), according to the manufacturer’s protocol. cDNA was synthesized from 1 µg total RNA using random primers, avian myeloblastosis virus RT, and a PrimeScript RT-PCR kit (Takara Bio, Inc. Shiga, Japan). The primers used for RT-PCR and real-time RT-PCR analysis are shown in Table 1. PCR was performed using Quick HS Taq DyeMix (Toyobo). PCR products were separated on a 1.5% agarose gel and stained with ethidium bromide. As an internal control for RNA quantity, the same cDNA was amplified using primers specific for rat actin mRNA. The intensity of bands was quantified by densitometry using ImageJ software (NIH). Real-time RT-PCR reactions were performed using a TaqMan gene expression assay kit or SYBR Premix Ex Taq (Takara Bio, Inc. Shiga, Japan) with a StepOnePlus real-time PCR system (Applied Biosystems, Foster City, CA). Reactions were conducted in a 10 µl reaction mixture and were incubated 1 minute at 95°C, followed by 40 cycles of a two-step amplification procedure composed of annealing/extension for 34 seconds at 60°C and denaturation for 5 seconds at 95°C. mRNA levels were quantified using a standard curve generated with serially diluted cDNA and normalized to *Gapdh* expression. A commercially available probe-primer set (Applied Biosystems) with proprietary sequences was used in PCR reactions for *csf-1r* (c-fms).

**Table 1:** List of primers used for RT-PCR and real-time RT-PCR.

Target gene	Forward primer	Reverse primer
*calcr* (calcitonin receptor)	5′-GCTGCTCTGATTGCTTCCAT-3′	5′-TTGTAGTAGACGGCACGAGT-3′
*CCR1*	5′-ACTGGTGAGCACTGTGATGC-3′	5′-TCAAGGTTCAAGGTCCCAAC-3′
*CCR2*	5′-CTTGTGGCCCTTATTTTCCA-3′	5′-AGATGAGCCTCACAGCCCTA-3′
*CCR5*	5′-CTGAGAAGGCTGGGAACAAG-3′	5′-GGCCTTGACCATTCTCTTCA-3′
*c-fos*	5′-CAGAAGGGGCAAAGTAGAGC-3′	5′-GCCTAGATGATGCCGGAAAG-3′
*ctsk* (cathepsin K) for RT-PCR	5′-ACCTTCGCGTTCCTTCAGTA-3′	5′-CACATTATCACGGTCGCAGT-3′
*ctsk* (cathepsin K) for real-time RT-PCR	5′-AGACGCTTACCCGTATGTGG-3′	5′-GGACACAGAGACGGGTCCTA-3′
*emr1* (F4/80)	5′-CAGCTGTCTTCCCGACTTTC-3′	5′-TAATCAAGATTCCGGCCTTG-3′
*itgam* (CD11b)	5′-CATCACCGTGAGTTCCACAC-3′	5′-GAGAACTGGTTCTGGCTTGC-3′
*nfatc1*	5′-TCATCGACTGTGCTGGGATC-3′	5′-CAACCCAAGTCTCACCACAG-3′
*tm7sf4(DC-STAMP)*	5′-TTGTGGAGGAACCAAAGAGG-3′	5′-AGGCTTACCGAAAGGAGAGC-3′
*tnfrs11a* (RANK)	5′-GCTTGCAGTAGTCTCAGTGG-3′	5′-AATCCACCATGCTTTCCGTC-3′
*β-*actin	5′-GACCCTGAAGTACCCCATTG-3′	5′-TTGCCGATAGTGATGACCTG-3′
*gapdh*	5′-TGCACCACCAACTGCTTAG-3′	5′-GGATGCAGGGATGATGTTC-3′

### Immunochemistry and Immunofluorescence of *in vitro* Culture

Cells were stained for Kat1 as described [16]. Briefly, cells were incubated with Kat1 mAb for 30 minutes and then fixed with 4% paraformaldehyde, and blocked in 3% normal goat serum. For immunochemistry, the cells were incubated with biotin-conjugated anti-mouse IgM, and detected using an ABC-AP kit (Vector Laboratory) according to the manufacturer’s protocol. For immunofluorescence of double staining with Kat1 and c-fms, cells were first stained with mouse mAb Kat1, then were stained with rabbit anti-c-fms antibody, and then were incubated with secondary antibodies; goat Alexa Fluor 568-conjugated anti-mouse IgG and Alexa Fluor 488-conjugated anti-rabbit IgG for 60 minutes. For double-staining with Kat1 and CD11b/c, cells were stained with Kat1, followed by incubating with goat Alexa Fluor 568-conjugated anti-mouse IgG; then, following washing, cells were incubated with mouse Alexa Fluor 488-conjugated anti-rat CD11b/c antibody. Nuclei were stained with To-Pro-3 iodide (Molecular Probes). The cells were examined with a Carl Zeiss LSM 5 Pascal confocal laser scanning microscope (Hyderberg, Germany). The numbers of Kat1^+^, c-fms^+^, CD11b/c^+^ cells or total cells were analyzed by LSM5Pascal and Zeiss Image Browser. Positive staining was counted in 10 randomly selected high-power fields under a microscope.

### Flow Cytometry

FACS analysis was performed as described with some modification [21]. NABMCs were cultured in the presence of M-CSF (3 ng/ml), TNF*-*α (20 ng/ml), and TGF-β (1 ng/ml) for 2 days. The cells were detached from the dish with 0.02% EDTA in PBS, and blocked with anti-FcR CD16/32 antibody (eBioscience) for 60 minutes. The cells were then incubated with biotin-conjugated Kat-1 mAb for 30 minutes. After three washes with α-MEM, 1% FCS, 10 mM EDTA, the cells were incubated with FITC-conjugated streptavidin (eBioscience). Immunofluorescence was analyzed using a FACSCalibur analyzer (Becton Dickinson). Irrelevant mouse biotin-labeled IgM antibody (Beckman) was included as a control.

### Panning of Kat^+^ Cells

NABMCs were cultured in the presence of M-CSF (3 ng/ml), TNF*-*α (20 ng/ml), and TGF-β (1 ng/ml) for 2 days. The cells were detached from the dish with 0.02% EDTA in PBS, and Kat1 mAb was added to the cells. Preosteoclasts coated with Kat1 mAb were then added to a petri dish coated with anti-mouse IgG and incubated at 4°C for 60 minutes with gentle mixing. After non-bound cells were recovered, cells bound to the coated dishes were isolated by flushing out using a pipette. The cells were then seeded in 96-well plates at a density of 1×10^5^ cells/well, and incubated for 2 days in the presence of M-CSF (3 ng/ml) and RANKL (30 ng/ml) to form MNCs.

### Cell Migration Assays

Chemotaxis assays were performed using a transwell chamber (pore size, 8 µm, Becton Dickinson Labware, NJ) as described with some modification [28]. NABMCs were cultured in the presence of TNF*-*α together with TGF-β for 3 days. The cells were detached from the dish, and suspended in PBS containing 0.1% BSA. The cells were loaded into the upper side of 24-well transwell inserts, and then placed above lower chambers containing various concentrations of MCP-1 or MIP-1α in α -MEM with 10% FCS. Cells were allowed to migrate for 4 hours at 37°C followed by fixation in 4% formaldehyde and staining with hematoxylin. The number of cells that migrated to the lower side of the filter was determined by microscopy.

### Induction of Adjuvant Arthritis (AA) in Rats

Female Lewis rats (Kyudo, Tosu, Japan) at 5 weeks old were, sedated under deep ether anesthesia, and intradermally injected at the base of the tail with complete adjuvant consisting of 25 mg/kg heat-killed *Mycobacterium butyricum* suspended in mineral oil as described [28]. The rats were housed in a 12-hour light/dark cycle with free access to water and chow. After twenty days, arthritis was assessed by observation of swelling.

### Immunohistochemistry of Bone Tissues of Rats

Before harvesting synovial tissues, living osteoclasts were stained *in situ* by direct injection of Kat1 mAb into rats as described [17]. Briefly, the ammonium sulfate-precipitated fraction of Kat1 mAb ascites was injected intraperitoneally into rats. Twelve hours after the injection, the animals were perfused through the left ventricle with 4% paraformaldehyde followed by dissection of the hind paw (tarsal bones and tibia). After further fixation overnight at 4°C, bone tissues were decalcified by treatment with EDTA for 3 weeks at 4°C with gentle shaking. After washing with PBS, tissue blocks were immersed in 5% sucrose/PBS, 4°C for 4 hours, followed by immersion in 10% sucrose/PBS, 4°C for 4 hours. These blocks were further immersed in 20% sucrose/PBS, 4°C overnight and embedded in Tissue-Tek O.C.T compound (Sakura, Tokyo, Japan). Frozen 12 µm sections were prepared using a cryostat HM560E (Microtome, Thermo Fisher Scientific, Walldorf, Germany). For double staining with c-fms antibody, sections were washed in PBS 3 times and incubated with anti-c-fms antibody, followed by secondary antibodies; Alexa Fluor 568-conjugated goat anti-mouse IgG and Alexa Fluor 488-conjugated goat anti-rabbit IgG. The cells were examined with a Carl Zeiss LSM 5 Pascal confocal laser scanning microscope.

## Results

### TNF-α in Combination with TGF-β induces Preosteoclasts at a Low Concentration of M-CSF in Rat Bone Marrow Culture Depleted of Stromal Cells

**Figure 1:**
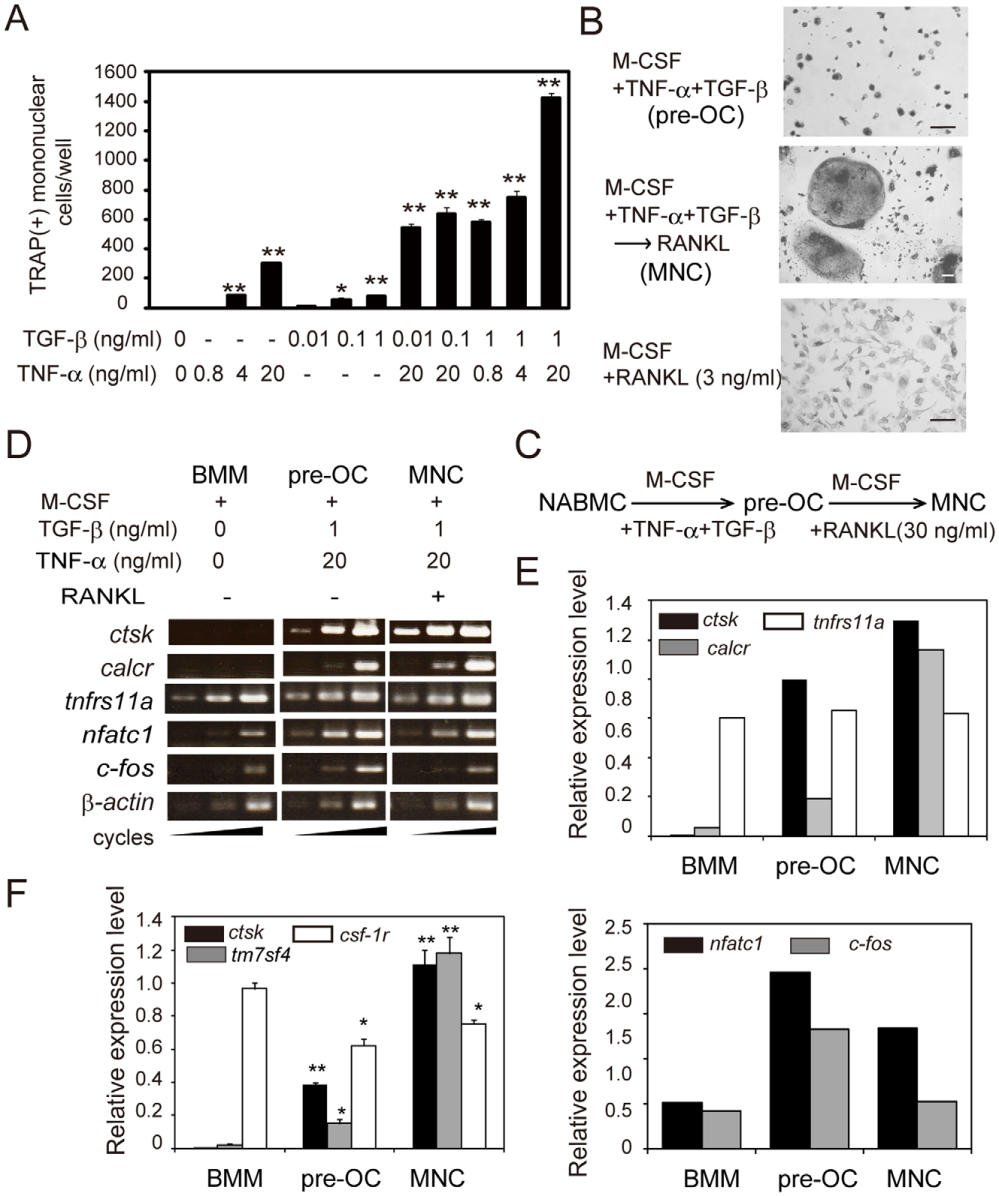
TNF-α in combination with TGF-β induces preosteoclasts at a low concentration of M-CSF. (A) Induction of TRAP^+^ mononuclear cells in the presence of various concentrations of TNF-α or TGF-β in the presence of M-CSF (3 ng/ml). Each value represents the mean ± SEM of three cultures. (B) Representative photomicrograph of TRAP^+^ mononuclear cells induced by M-CSF (3 ng/ml), TNF-α (20 ng/ml), and TGF-β (1 ng/ml) (top); or TRAP^+^ MNCs induced from preosteoclasts by RANKL (30 ng/ml) as illustrated in (C) (middle); or TRAP^+^ mononuclear cells induced by RANKL (3 ng/ml) and M-CSF (10 ng/ml) (bottom). Bar = 50 µm. (C) Illustration of sequence of culture conditions. (D) RT-PCR analysis of mRNA expression of osteoclast-specific genes and genes encoding cell surface proteins in cultures of BMM, preosteoclasts (pre-OC), and MNCs. (E) Relative expression levels of *ctsk, calcr, tnfrs11a* (RANK), *nfatc,* and *c-fos* were normalized with β*-actin*. (F) Real-time RT-PCR analysis of expression levels of *ctsk, tm7sf4* (DC-STAMP), and *csf-1r* (c-fms). Expression levels were normalized with *gapdh*. NABMCs were cultured for 3 days in the presence of various concentrations of TNF-α, TGF-β, M-CSF, and RANKL. To induce TRAP^+^ MNCs, RANKL was added to preosteoclasts induced by treatment with TNF-α, TGF-β, and M-CSF, and cultures were continued for 2 days. At the end of culture, TRAP-staining was performed and TRAP^+^ mononuclear cells were counted. Total RNA from cells was prepared and analyzed by semi-quantitative RT-PCR or real-time RT-PCR using specific primers as in *Materials and Methods.* Each value represents the mean ± SEM (n = 3). Statistical significance was determined by Student’s *t* test; *P<0.05, **P<0.01 compared with BMM. Experiments were performed three times using three different rats, and similar results were obtained.

We first examined whether either TNF*-*α or TGF-β alone, or TNF*-*α together with TGF-β induced mononuclear osteoclast precursor cells at various concentrations of M-CSF. At 3 ng/ml M-CSF, TNF*-*α or TGF-β alone did not markedly induce the formation of TRAP^+^ mononuclear cells (Fig. 1A). However, TNF*-*α together with TGF-β induced significantly greater formation of TRAP^+^ mononuclear cells, but did not induce the formation of TRAP^+^ multinucleated cells (MNCs) (Fig. 1A, and B top). In this culture, the intensity of TRAP staining was strong, and the percentage of TRAP^+^ mononuclear cells in total cells was high (Fig. 1B, top). Addition of RANKL (30 ng/ml) 2 days after addition of TNF*-*α, TGF-β, and M-CSF (3 ng/ml) to the culture induced the formation of TRAP^+^ MNCs (Fig. 1B, middle), indicating that TRAP^+^ mononuclear cells are osteoclast precursor cells. Various concentrations of RANKL and M-CSF were tested in rat bone marrow cultures, and we determined the concentrations that induced preosteoclasts but not MNCs. A low concentration of RANKL (3 ng/ml) in the presence of M-CSF (10 ng/ml) also induced the formation of TRAP^+^ mononuclear cells but not TRAP^+^ MNCs; however, the culture included a number of TRAP^−^ mononuclear cells (Fig. 1B, bottom). These results suggest that TNF*-*α and TGF-β efficiently induce an enriched population of mononuclear osteoclast precursor cells in rat bone marrow cultures at a low concentration of M-CSF (3 ng/ml). At a higher concentration of M-CSF (30 ng/ml), TNF*-*α together with TGF-β induced formation of TRAP^+^ MNCs (data not shown).

To analyze characteristics of mononuclear osteoclast precursor cells induced by TNF*-*α and TGF-β, we compared expression of several osteoclast-specific genes and genes encoding a number of cell surface proteins in the culture of osteoclast precursor cells with that in the cultures of BMM and MNCs by semi-quantitative RT-PCR analysis (Fig. 1D and E). Treatment with TNF*-*α together with TGF-β strongly increased the expression of not only cathepsin K (*ctsk*), but also calcitonin receptor (*calcr*), NFATc1 (*nfatc1*), and *c-fos* compared to BMM. As shown in Fig. 1F, real-time RT-PCR analysis showed that the expression of *ctsk* and DC-STAMP (*tm7sf4*) was significantly increased in preosteoclasts compared to BMM and further increased in MNCs. On the other hand, c-fms (*csf-1r*) and RANK (*tnfrsf11a*) were expressed in the cultures of preosteoclasts at almost the same level as BMM, but expression of *csf-1r* was decreased in MNCs (Fig. 1E and F). These results demonstrate that TRAP^+^ mononuclear cells induced by TNF*-*α and TGF-β are mononuclear osteoclasts, i.e., preosteoclasts.

### Characterization of Preosteoclasts with an Osteoclast-specific mAb Kat1

**Figure 2:**
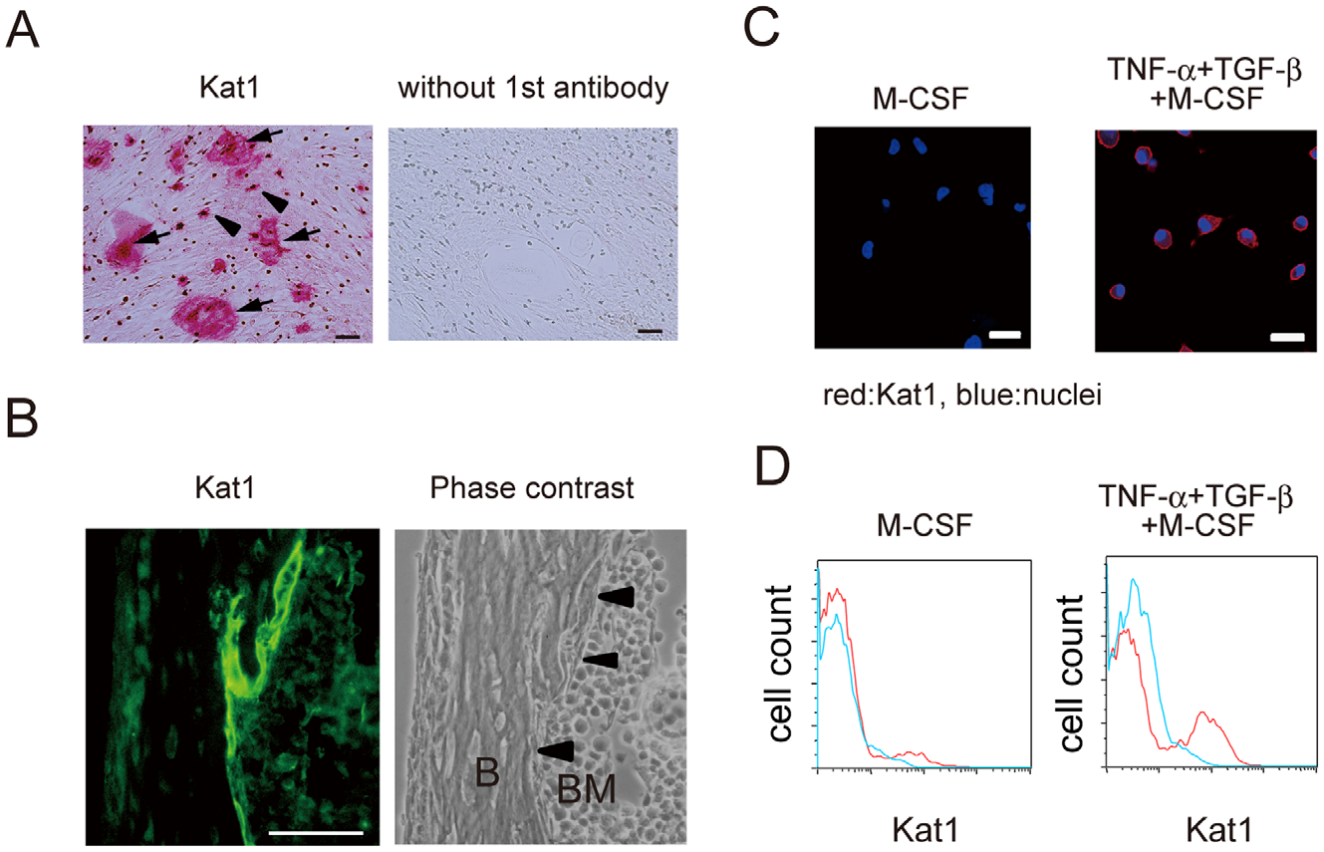
Treatment with TNF-α and TGF-β specifically induces the formation of Kat1^+^ preosteoclasts. (A) Immunostaining image of Kat1 antigen (red) in rat bone marrow cultures. Arrows: multinucleated osteoclasts. Arrow heads: osteoclast precursors. Bar = 50 µm. Rat bone marrow cells were cultured in the presence of 1,25(OH)_2_D_3_ for 6 days, and were then stained with Kat1 mAb. (B) Immunofluorescence image of stained Kat1 antigen (green) in rat mandible. Living osteoclasts were stained *in situ* by the direct injection of the Kat1 mAb; rats were perfused with 4% paraformaldehyde followed by dissection of mandible, and sections were prepared as in *Materials and Method.* Tissue sections were stained with FITC-conjugated anti-mouse IgM and observed by fluorescence microscopy. Arrow heads: osteoclasts. Right panel; phase-contrast view of same field as left panel. B: bone. BM: bone marrow. Bar = 50 µm. NABMC were cultured in the presence of various concentrations of M-CSF, TNF-α, and TGF-β for 2 days (C, D). (C) Cells were stained for Kat1 and analyzed by confocal microscopy. Immunofluorescence images of Kat1 antigen (red) and nuclei (blue) in cells induced by M-CSF alone (left), or TNF-α (20 ng/ml), TGF-β (1 ng/ml), and M-CSF (right). Bar = 50 µm. (D) FACS analysis with Kat1 mAb (red) and control IgM (blue). Cells were induced by M-CSF alone (left), or TNF-α (20 ng/ml), TGF-β (1 ng/ml), and M-CSF (right).

To analyze preosteoclasts induced by TNF*-*α and TGF-β in detail, we next used Kat1 mAb which specifically recognizes osteoclasts and osteoclast precursor cells in rats. Kat1 stained osteoclasts formed in rat bone marrow culture *in vitro*. We have reported that viable osteoclasts can be detected *in situ* in mandible tissues of newborn rats by injecting into the peritoneal cavity of rats [17]. As described previously, osteoclasts formed *in vitro* and present on rat mandible bone *in vivo* were detected by Kat1 mAb (Fig. 2A and B). Analysis with confocal laser microscopy showed that TNF*-*α and TGF-β induced the formation of Kat1^+^ mononuclear cells in the presence of M-CSF, and Kat1 antigen was localized on the cell membrane of preosteoclasts (Fig. 2C, right). In contrast, Kat-1^+^ cells were not detected in BMM (Fig. 2C, left). In addition, FACS analysis also showed that Kat1 antigen is expressed on the cell surface of preosteoclasts induced by TNF*-*α and TGF-β, but is not expressed on BMM (Fig. 2D).

**Figure 3:**
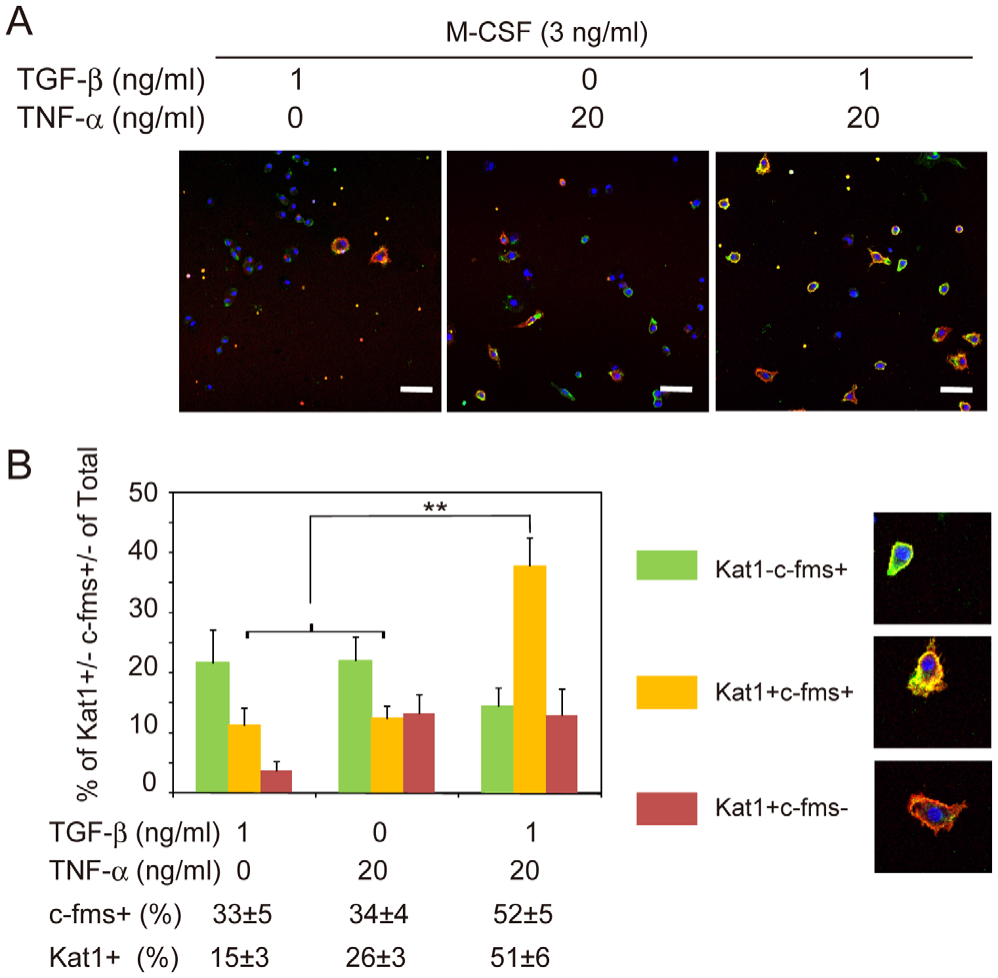
TNF-α and TGF-β specifically increase subpopulations of Kat1^+^c-fms^+^ and Kat1^+^c-fms^−^ preosteoclasts. (A) Confocal microscopic analysis of Kat1^+^, c-fms^+^, or Kat1^+^c-fms^+^ cells in cultures induced by M-CSF and various concentrations of TNF-α, and TGF-β. Bar = 50 µm. (B) Percentages of Kat1**^−^**c-fms^+^, Kat1^+^c-fms^+^ or Kat1^+^c-fms**^−^** cells among cells induced by M-CSF and various concentrations of TNF-α and TGF-β. NABMCs were cultured for 2.5 days. Cells were stained for Kat1 and c-fms, and analyzed by confocal microscopy with LSM5Pascal software. The photographs show typical cultures. Each value represents the mean ± SEM of ten fields in a typical culture. Statistical significance was determined by Student’s *t* test; **P<0.01 compared with TNF-α or TGF-β alone. We have repeated these experiments and similar results were obtained.

To analyze the population of preosteoclasts induced by TNF-α and TGF-β in more detail, we next performed double staining of cells with Kat1 and anti-c-fms antibodies. TNF-α alone increased the population of single-positive Kat1^+^c-fms^−^cells in total cells compared to those induced by TGF-β alone. But TNF-α and TGF-β increased the population of double-positive Kat1^+^c-fms^+^ cells in total cells compared to those induced by TNF-α or TGF-β alone (Fig. 3A and B). In contrast, treatment with TNF-α and TGF-β did not increase the formation of Kat1^−^c-fms^+^ cells. Thus, TNF*-*α combined with TGF-β preferentially generated subpopulations of Kat1^+^ preosteoclasts, especially Kat1^+^c-fms^+^ cells.

### Analysis of the Differentiation Status of Preosteoclast Subpopulations Induced by Various Combinations of TNF-α and TGF-β

**Figure 4:**
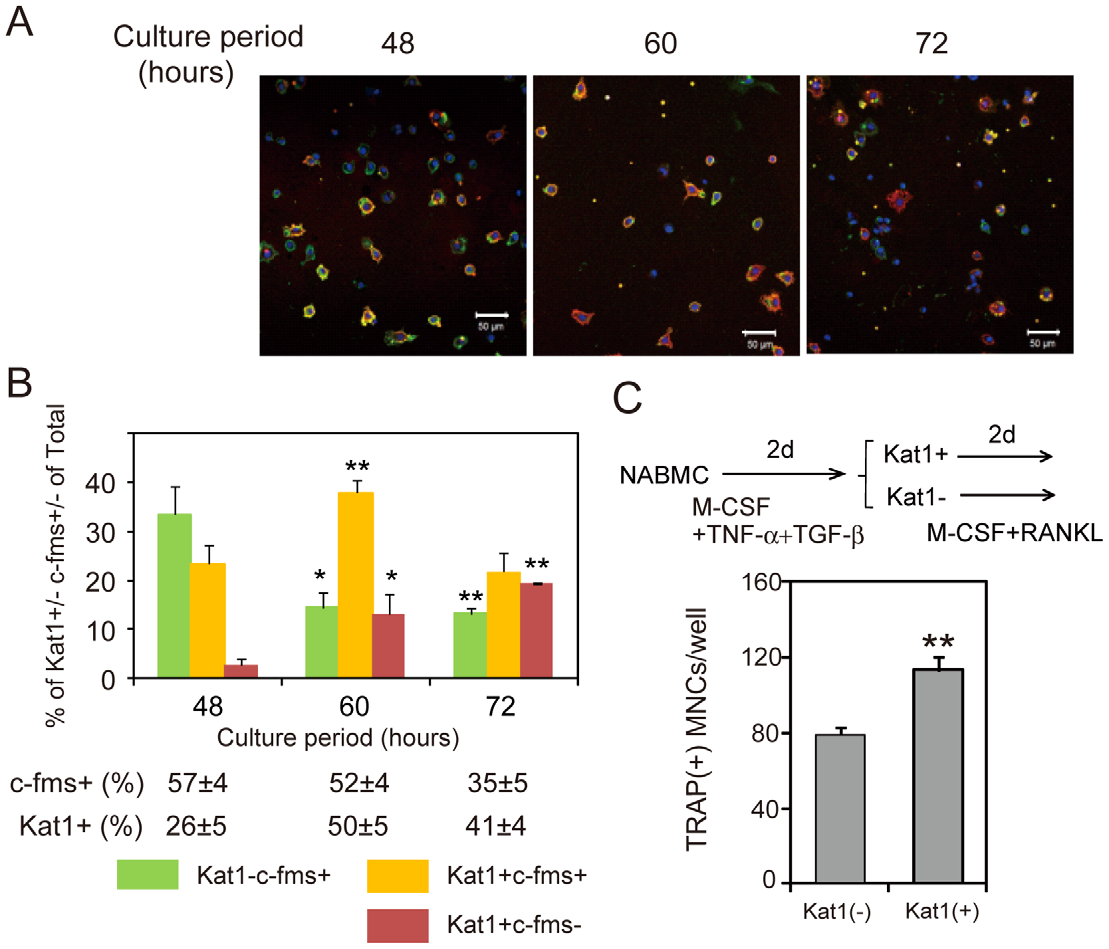
Kat1^+^ cells represent a late differentiation stage of preosteoclasts. (A) Photographs of cells that were Kat1**^−^**c-fms^+^, Kat1^+^c-fms^+^, or Kat1^+^c-fms**^−^** in time-course cultures. Cells were stained for Kat1 and c-fms, and analyzed by confocal microscopy with LSM5Pascal software. Bar = 50 µm. (B) Distribution of cells from cultures shown in (A). Each value represents the mean ± SEM of ten fields in a typical culture. Statistical significance was determined by Student’s *t* test; *P<0.05, **P<0.01 compared with 48 hours. (C) Formation of TRAP^+^ MNCs from enriched populations of Kat1^+^ or Kat1^−^ cells in preosteoclasts cultures. NABMCs were cultured with TNF-α, TGF-β, and M-CSF for 2.5 days. Kat1^+^ cells and Kat1^−^ cells in preosteoclasts cultures were isolated by panning with mAb Kat1 as described in *Materials and Methods*, and cultured for 2 days in the presence of RANKL (30 ng/ml) and M-CSF (3 ng/ml). Statistical significance was determined by Student’s *t* test. **P<0.01 compared with Kat-1^−^ cells. We have repeated this experiments and similar results were obtained.

To analyze differentiation stages of preosteoclast subpopulations, we performed a time course analysis of cell surface expression of Kat1 antigen and c-fms using the cell population induced by TNF-α (20 ng/ml) and TGF-β (1 ng/ml) (Fig. 4A). Kat1^−^c-fms^+^ cells appeared in the early stage (48 hours) of culture and their numbers decreased thereafter (Fig. 4B). The percentages of Kat1^+^c-fms^+^ cells increased from 48 to 60 hours of culture, and decreased by 72 hours of culture. In contrast, Kat1^+^c-fms^−^ cells had not appeared by 48 hours but were present at 60 hours of culture, and the percentage was not changed at 72 hours of culture (Fig. 4B). These data indicate that Kat1^−^c-fms^+^ cells are immature osteoclast precursors, and that Kat1^+^ cells are more differentiated cells. The data also suggest that Kat1^+^c-fms^−^ cells may be more differentiated compared to Kat1^+^c-fms^+^ cells. To assess osteoclast differentiation capacity of Kat1^+^ cells, we enriched Kat-1^+^ cells from preosteoclasts cultures induced by TNF-α and TGF-β, and compared osteoclast formation induced by RANKL from Kat1^+^ cells to that from Kat1^−^ cells. Significantly higher numbers of TRAP^+^ MNCs were formed from Kat1^+^ cells, although a substantial number of TRAP^+^ MNCs were formed from Kat-1^−^ cells (Fig. 4C).

**Figure 5:**
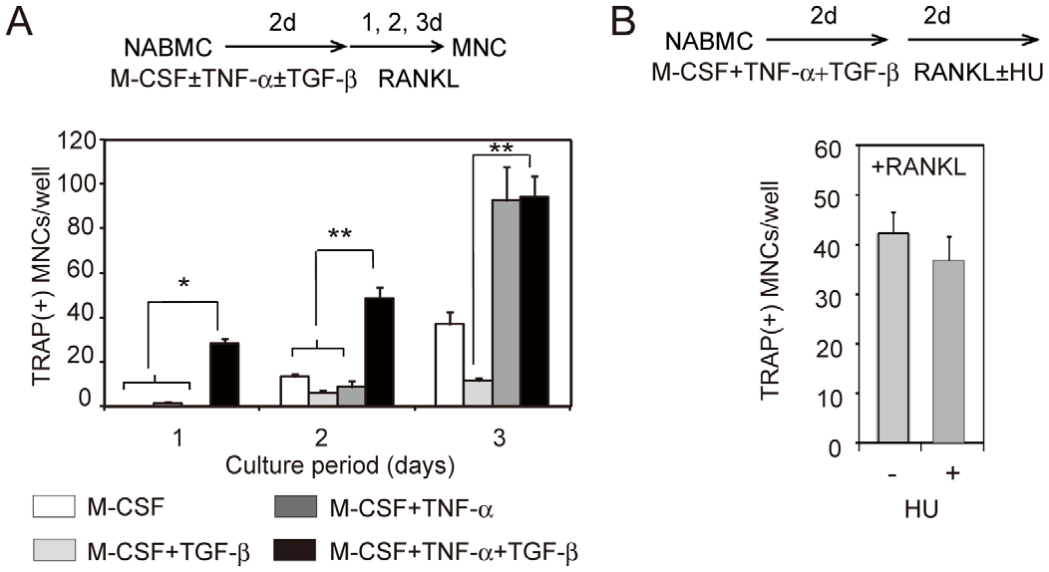
Analysis of differentiation potentials of osteoclast precursor cells induced by various conditions. (A) Time course of formation of TRAP^+^ MNCs from mononuclear cells induced by M-CSF in combination with TNF-α and TGF-β separately or together. (B) Effect of hydroxyurea (HU) on RANKL-induced formation of TRAP^+^ MNCs from preosteoclasts. NABMCs were cultured with TNF-α, TGF-β, and M-CSF for 2 days, and were then cultured further for 1–3 days (A) in the presence of RANKL (30 ng/ml) and M-CSF (3 ng/ml), or (B) in the presence or absence of HU (100 µM). Each value represents the mean ± SEM of three cultures. Statistical significance was determined by Student’s *t* test; *P<0.05, **P<0.01.

We further analyzed the differentiation ability of the preosteoclasts induced by TNF-α and TGF-β. As shown in Fig. 1B, after formation of preosteoclasts by TNF-α and TGF-β, the addition of RANKL to the culture generated TRAP^+^ MNCs. We then analyzed the time course of RANKL-mediated formation of TRAP^+^ MNCs from cells induced by various conditions (Fig. 5A). TRAP^+^ MNCs were generated from preosteoclasts induced by TNF-α plus TGF-β within 1 day after addition of RANKL. In contrast, the number of TRAP^+^ MNCs generated from BMM or cells induced by TNF-α alone was increased at 3 days after addition of RANKL. In addition, hydroxyurea, which inhibits DNA synthesis, did not inhibit the formation of TRAP^+^ MNCs from preosteoclasts induced by TNF-α plus TGF-β (Fig. 5B). These data suggest that cells induced by TNF-α and TGF-β, which are mainly Kat1^+^c-fms^+^, are terminally differentiated preosteoclasts and are poised for fusion.

### Analysis of Expression of Surface Proteins in Preosteoclasts and MNCs

**Figure 6:**
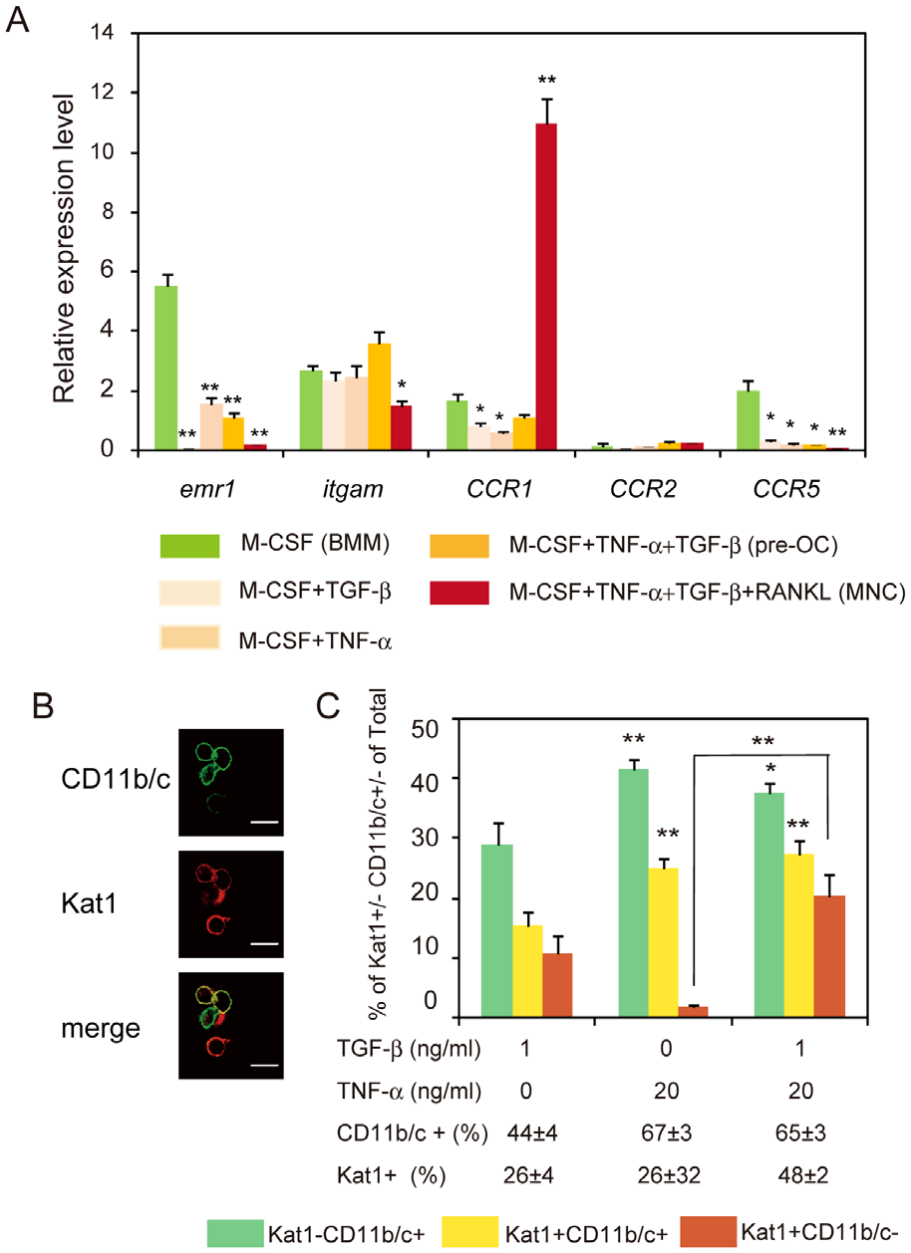
Analysis of expression of genes encoding cell surface proteins in BMM, preosteoclasts, and MNCs. (A) Relative mRNA expression levels of *emr1* (F4/80), *itgam* (CD11b), *CCR1, CCR2,* and *CCR5* in cultures of BMM, preosteoclasts (pre-OC) and MNCs. (B) Immunofluorescence images of preosteoclasts stained for Kat1 antigen (red) and CD11b/c (green). (C) Percentages of Kat1**^−^**CD11b/c^+^, Kat1^+^CD11b/c^+^ or Kat1^+^CD11b/c**^−^** cells among cells induced by M-CSF in combination with TNF-α and/or TGF-β. NABMCs were cultured for 3 days in the presence of various combinations of TNF-α, TGF-β, and M-CSF to induce formation of BMM or preosteoclasts. After formation of preosteoclasts, RANKL was added to induce formation of MNCs. Total RNA from cells was prepared and analyzed by real-time RT-PCR using specific primers as in *Materials and Methods*. Expression levels were normalized with *gapdh* (A). The cells were stained for Kat1 and CD11b and analyzed by confocal microscopy using LSM5Pascal software. The photographs show typical cultures. (B). Each value represents the mean ± SEM (n = 3, A) or (cell counts from ten-microscope fields, C). Statistical significance was determined by Student’s *t* test; *P<0.05, **P<0.01 compared with BMM (A) or TGF-β alone (C). Bar = 20 µm. The experiments were performed three times, and similar results were obtained.

To characterize preosteoclasts induced by TNF*-*α and TGF-β in more detail, we used real-time RT-PCR analysis to compare expression levels of several genes encoding surface proteins among cells induced by various conditions (Fig. 6A). The expression of *emr1* (F4/80) a macrophage marker was decreased in preosteoclasts induced by TNF-α plus TGF-β. Treatment with TGF-β alone resulted in markedly reduced expression of *emr1* in comparison with BMM. On the other hand, no significant difference was seen in the mRNA expression levels of the monocyte marker, *CD11b* (*itgam*) between preosteoclasts and BMM (Fig. 6 A). The expression level of *itgam* was rather decreased in MNCs. Using an antibody against rat CD11b/c, we further analyzed the population of preosteoclasts by double staining of cells with Kat1 and CD11b/c antibodies. TNF-α alone increased the population of Kat1^−^CD11b/c^+^ and Kat1^+^CD11b/c^+^ cells, but the number of Kat1^+^CD11b/c^−^ cells induced in total cells was markedly less compared to number induced by TGF-β alone. Addition of TGF-β to TNF-α increased the population of Kat1^+^CD11b/c^−^ cells in total cells compared to those induced by TNF-α alone (Fig. 6B and C). Thus, Kat1^+^CD11b/c^+^ cells were induced by TNF-α and TGF-β, but also TNF-α alone.

To examine the expression of surface proteins of preosteoclasts in more detail, we analyzed the expression of chemokine receptors *CCR1, CCR2,* and *CCR5,* which are involved in osteoclastogenesis. The expression of *CCR1* was decreased in cells treated with TNF-α or TGF-β alone, and was not up-regulated in preosteoclasts. Interestingly, however, CCR1 expression was markedly increased in MNCs induced by RANKL (Fig. 6A). On the other hand, the expression of *CCR2* was low, and was not changed among BMM, preosteoclasts or MNCs. In contrast, *CCR5*, which was highly expressed in BMM, was decreased in preosteoclasts and MNCs. Thus, using preosteoclast cultures induced by TNF-α and TGF-β, it was found that *itgam*, CCR1, and CCR2 were neither down-regulated nor up-regulated in preosteoclasts, but expression of *emr1* and CCR5 were down-regulated in preosteoclasts. In addition, comparison of preosteoclasts and MNCs showed that CCR1 expression was markedly increased in the multinucleation step during osteoclastogenesis.

### MIP-1α and MCP-1 Promote RANKL-induced Multinucleation of Preosteoclasts

**Figure 7:**
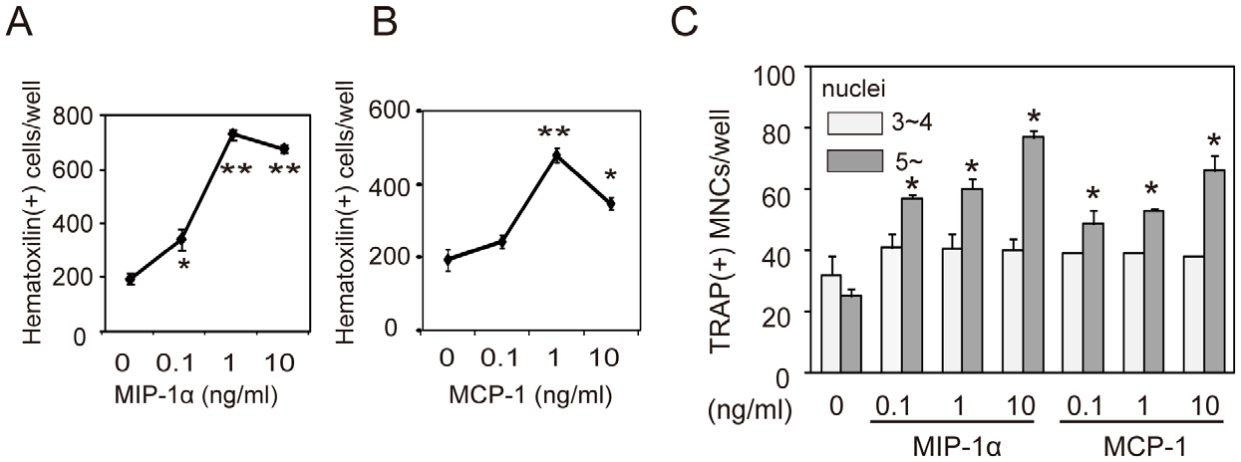
Chemotaxis of preosteoclasts toward chemokines and effect of chemokines on multinucleation of preosteoclasts. Chemotaxis of preosteoclasts toward chemokine MIP-1α (A) and MCP-1 (B). (C) Effect of MIP-1α or MCP-1 on the formation of TRAP^+^ MNCs from preosteoclasts in the presence of RANKL and M-CSF. NABMCs were cultured in the presence of TNF-α, TGF-β, and M-CSF. The cells were detached and cultured in a transwell. Preosteoclasts were added to the upper chamber of the transwell, and various concentrations of MIP-1α or MCP-1 were added to the lower chamber (A, B). After 4 hours of culture, migration of cells to the lower side of the filter was analyzed. Preosteoclasts induced by TNF-α, TGF-β, and M-CSF were then cultured in the presence of RANKL, M-CSF, and various concentrations of MIP-1α or MCP-1 for 2 days (C). TRAP^+^ cells having three or four, or more than five nuclei were counted as MNCs. Each value represents the mean ± SEM of three cultures. Significance was determined by Student’s *t*-test; *P<0.05 compared to cultures without chemokines.

The results from expression analysis of chemokine receptors CCR1 and CCR2 in preosteoclasts suggested that preosteoclasts would exhibit chemotactic activity toward the chemokine ligands, MIP-1α and MCP-1. To asses this possibility, we examined migration activity of preosteoclasts toward MIP-1α and MCP-1. Cell migration toward MIP-1α and MCP-1 increased in a dose-dependent fashion (Fig. 7A and B). To determine if CCR1 and CCR2 were involved in multinucleation from preosteoclasts, we analyzed the effect of MIP-1α and MCP-1 on formation of TRAP^+^ MNCs from preosteoclasts in the presence of RANKL and M-CSF. Both MIP-1α and MCP-1 significantly stimulated RANKL-induced fusion of preosteoclasts, specifically inducing large multinucleated cells having more than 5 nuclei (Fig. 7C).

### Kat^+^c-fms^+^ Preosteoclasts are Present in Bone Tissues of AA Rats

**Figure 8:**
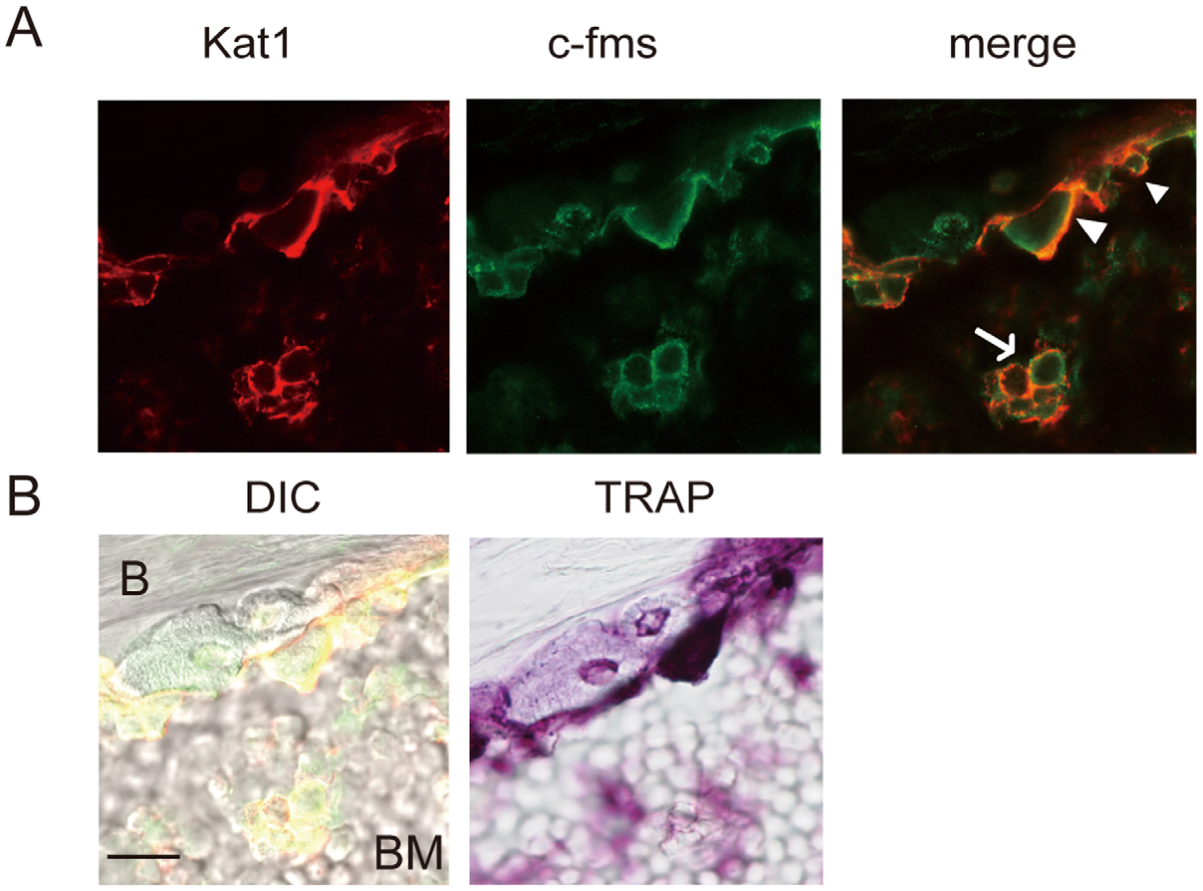
Kat1^+^c-fms^+^ cells are present in bone tissues obtained from AA rats. Immunofluorescence images of Kat1 antigen (red) and c-fms (green) (A), and different interference contrast (DIC) and TRAP staining (B) of bone tissues obtained from ankle joint of AA rats are shown. Living osteoclasts were stained *in situ* by the direct injection of the Kat1 mAb, and rats were perfused with 4% paraformaldehyde followed by dissection of the hind paw. Frozen sections were prepared as in *Materials and Methods.* Tissue sections were then stained with c-fms antibody, followed by treating with Alexa Fluor 568-conjugated goat anti-mouse IgG and 488-conjugated anti-rabbit IgG. The same section was also stained for TRAP. B, bone; BM, bone marrow. Bar = 20 µm. Arrow heads and arrow indicate Kat1^+^c-fms^+^ cells.

To determine whether Kat1 and c-fms double positive cells were present *in vivo*, we performed Kat1 mAb *in situ* immunostaining of bone tissues obtained from ankle joints of AA rats. We first injected Kat1 mAb into AA rats, and later immunostained bone tissues with anti-c-fms antibody or stained for TRAP. Double–positive cells were detected in the site of bone destruction in distal tibia. Kat1^+^ cells present in the bone surface also expressed c-fms (Fig. 8A, arrow heads), and were also positive for TRAP (Fig. 8B). In the bone marrow area, one pair of cells positive for both Kat1 antigen and c-fms can be seen (Fig. 8A, arrow). These data demonstrate the presence of Kat1^+^c-fms^+^ cells in bone tissue accompanying inflammatory bone destruction.

## Discussion

Although the molecular mechanisms regulating osteoclast differentiation from hematopoietic precursors and the essential role of RANKL has been well described, relatively little is known about characteristics and subpopulations of osteoclast precursor cells and preosteoclasts. Bone marrow cells and BMM are widely utilized to form osteoclasts by treatment with 1,25(OH)_2_D_3_ or RANKL *in vitro* [29]. However, because most *in vitro* culture systems induce osteoclasts without arresting cells at the stage of preosteoclasts, it is difficult to investigate the characteristics of osteoclast precursors and preosteoclasts. In addition, most such cultures include large numbers of undifferentiated cells and cells of lineages unrelated to osteoclasts. We also found that the percentage of preosteoclasts in total cells was not increased by use of various concentrations of RANKL and M-CSF in rat bone marrow culture. Here, we demonstrate that the combination of TNF-α and TGF-β efficiently induces the formation of preosteoclasts, which express osteoclast-specific genes and the osteoclast-specific antigen, Kat1, and comprised 50–60% of total cells in the culture.

Osteoclast precursor cells, monocytes, and macrophages are heterogeneous populations. In mouse monocytes, there are classical inflammatory monocytes and non-classical monocytes that play different roles in inflammation [30]. The short-lived CCR2^+^Gr1^−^ subset is actively recruited to inflamed tissues, whereas the CCR2^−^Gr1^+^ subset is recruited to non-inflamed tissues in a CX_3_CR1-dependent manner. CD14 and CD16 are utilized to define human monocyte subsets, including the CD14^+^CD16^−^ and CD14^lo^CD16^+^ subsets, which have different roles and homing potentials [31]. Macrophages are also classified into M1 and M2 macrophages, which are functionally differentially polarized in response to microorganisms and host mediators [32]. It has been reported that some populations of monocytes have differential potentials to differentiate into osteoclasts. The CD16^−^ human blood monocyte subset, but not the CD16^+^ monocyte subset, differentiates into osteoclasts when treated with RANKL and M-CSF [33]. However, it is still poorly understood that among preosteoclasts–more differentiated osteoclast precursor cells–there may be distinct subsets (phenotypic diversity). Cell surface markers such as c-fms, RANK, and CD11b have been used to identify mouse osteoclast precursor cells, but these markers are also expressed on macrophages. To characterize preosteoclasts in details, it is necessary to use unique cell surface markers to characterize them. Therefore, we used Kat1 mAb which specifically detects cells of the osteoclast lineage. The Kat1 antigen is different from the calcitonin receptor and RANK, but its distribution is similar to that of the calcitonin receptor [17], [18], indicating that Kat1^+^ cells are in a more differentiated stage. In this study, we have shown that combined use of c-fms or CD11b along with Kat1 mAb was useful for analyzing preosteoclasts.

Using Kat1 antibody, c-fms^+^ cells were classified into Kat1^−^c-fms^+^ and Kat1^+^c-fms^+^ subpopulations. TNF-α and TGF-β preferentially increased expression of osteoclast-specific genes, and increased the number of Kat1^+^c-fms^+^ cells. The population of cells induced by TNF-α and TGF-β rapidly differentiates into multinucleated osteoclasts without DNA synthesis, suggesting that Kat1^+^c-fms^+^ cells represent a more differentiated population of osteoclast precursors. We also found that most Kat1^+^ cells were also positive for c-fms in bone tissues of AA rats, indicating that Kat1^+^c-fms^+^ cells are present *in viv*o. In addition, we found that Kat1^+^ cells could be divided into Kat1^+^c-fms^+^ and Kat1^+^c-fms^−^. Because the Kat1^+^c-fms^−^ subpopulation appeared later in osteoclastogenesis, Kat1^+^c-fms^−^ cells may be more a differentiated population than Kat1^+^c-fms^+^. Alternatively, because M-CSF is known to be survival factor, Kat1^+^c-fms^−^ cells may be apoptotic cells. Since the Kat1 antigen has not been identified, further characterization of the Kat1^+^ antigen and preosteoclasts populations is necessary.

Several reports indicate that osteoclast precursor cells express CD11b [15], [29]. In our study, preosteoclasts induced by TNF-α and TGF-β expressed CD11b at levels similar to BMM, and some populations of CD11b^+^ cells were Kat1^+^, confirming previous results. However, expression of CD11b decreased at the multinucleation step, suggesting that CD11b expression is decreased during maturation of osteoclasts. There were some differences in characteristics between Kat1^+^CD11b^+^ and Kat1^+^c-fms^+^ populations. First, in comparison with Kat1^+^c-fms^+^ cells which are selectively increased by TNF-α and TGF-β, Kat1^+^CD11b^+^ cells were also increased by TNF-α. In addition, the percentages of c-fms+ or CD11b^+^ cells among Kat1^+^ cells were 78.6±6.2 or 60.7±5.5, respectively. These data suggest that Kat1^+^c-fms^+^ may be more selective phenotype for preosteoclasts than Kat1^+^CD11b^+^. Second, TNF-α alone increased the population of Kat1^+^CD11b^+^ but not Kat1^+^c-fms^+^, and decreased the population of Kat1^+^CD11b^−^ cells. The results suggest that specific population of preosteoclasts may be induced in inflammatory conditions. TNF-α-transgenic mice developed erosive arthritis, and the number of osteoclasts precursors in blood and inflamed joints was increased. [34], [35]. In the transgenic mice, TNF-α increased the number of CD11b^high^ osteoclast precursor cells, which may differ from normal monocytes, and may represent a specific population of osteoclast precursor cells. Consistent with these results, when we analyzed Kat1^+^CD11b^+^ cells in bone marrow cells of AA rat, the number of Kat1^+^CD11b^+^ cells was significantly increased in comparison to the numbers in control rats (A. Kukita *et al.* unpublished data). Because, TNF-α alone did not fully induce the differentiation into preosteoclasts *in vitro,* Kat1^+^CD11b^+^ cells may be immature osteoclast precursor cells. Further study will be necessary to elucidate the properties of Kat1^+^CD11b^+^ cells. Recently, *in vivo* imaging techniques made it possible to identify living cells *in vivo*. CD11b^+^ cells were used to track migration of osteoclast precursor cells from blood into bone [36]. Similar use of an osteoclast-specific antibody such as Kat1 in concert with c-fms or CD11b should give us more details of properties of osteoclast precursor cells accumulating in inflammatory disease sites.

In this study, we used TNF-α and TGF-β to induce preosteoclasts, but found that TNF-α and TGF-β have distinct roles in osteoclastogenesis. TNF-α alone induced a small number of TRAP-positive cells, which expressed osteoclast-specific genes and Kat1^+^CD11b^+^. In contrast, TGF-β did not efficiently induce osteoclastogenesis. However, TGF-β strongly inhibited the expression of *emr-1*. *Emr-1* encodes the F4/80 antigen, which is specifically expressed in macrophages. It has been shown that TGF-β stimulates or inhibits osteoclastogenesis depending on the *in vitro* culture system, and also promotes apoptosis of osteoclasts [37], [38]. Several recent reports have shown that TGF-β directly affects osteoclast signaling in cells of the osteoclast lineage, and stimulates osteoclastogenesis, particularly early in the differentiation pathways [39], [40]. Our data suggest that the stimulatory activity of TGF-β on osteoclastogenesis may also be mediated by inhibiting differentiation into macrophages.

Interestingly, we found that expression of chemokine receptors is regulated during differentiation from BMM to preosteoclasts and MNCs. Although expression of osteoclast-specific genes such as cathepsin K was up-regulated in preosteoclasts, expression of CCR1 was not up-regulated. However, CCR1 expression was markedly increased in MNCs induced by RANKL. CCR1, which is a receptor for MIP-1α, is up-regulated by RANKL, and is involved in the function of osteoclasts and cell-fusion of osteoclasts [10], [13], [41]. Recently, it has been reported that the CCR1 gene was a target of NFATc1 [41]. However, the timing of the induction of CCR1 in osteoclastogenesis is not clear. Our results show that the induction of CCR1 expression occurs during fusion. The results suggest that the therapeutic regulation of CCR1 expression and function may be useful for controlling bone resorption. Consistent with our data, it was very recently reported that CCR1 blockade efficiently blocked osteolysis in a mouse model of myeloma bone disease [42]. We also found that a CCR1 ligand, MIP-1α, was chemotactic for preosteoclasts, and stimulated fusion. CCR2, which is a receptor for MCP-1, and is abundantly expressed in macrophages, has recently been found to be up-regulated in CD11b^high^ osteoclast precursor cells [43]. Ablation of the CCR2 gene results in a defect in osteoclastogenesis. In our study, expression of CCR2 was low, and was not up-regulated in preosteoclasts and MNCs. However, preosteoclasts expressed CCR2, and MCP-1 was also chemotactic for preosteoclasts, and stimulated fusion, suggesting that CCR2 is also involved in osteoclastogenesis. On the other hand, the expression of *CCR5*, which was abundantly expressed in macrophages, was down-regulated during differentiation into preosteoclasts and osteoclasts, suggesting that CCR5 may not be involved in osteoclastogenesis.

In the present study, we show that a combination of TNF-α and TGF-β preferentially directs osteoclast differentiation and increases Kat1^+^c-fms^+^ preosteoclasts, which express several osteoclast-specific genes. In our comparison of preosteoclasts with BMM and osteoclasts, we found that preosteoclasts have unique characteristics. CD11b, CCR1, and CCR2 were expressed in preosteoclasts, but only CCR1 expression was markedly increased during fusion. The identification of preosteoclasts will be useful for investigating the origin and the trafficking of preosteoclasts formed in normal bone and in inflammatory disease. In addition, identification of surface proteins that are differentially expressed in preosteoclasts and MNCs may prove useful in controlling the fusion step in osteoclastogenesis and bone resorption.
